# Metformin and estrogen modulation in LABC with T2DM: A 36-month randomized trial

**DOI:** 10.1515/med-2025-1262

**Published:** 2025-10-06

**Authors:** Fengjia Liu, Chunhong Xu, Yingcheng Bai, Ying Yuan

**Affiliations:** No. 971 Hospital of the People’s Liberation Army Navy, Qingdao, Shandong, 266000, China

**Keywords:** metformin, locally advanced breast cancer, type 2 diabetes mellitus, estrogen levels, prognostic care

## Abstract

**Background:**

Patients with locally advanced breast cancer (LABC) and type 2 diabetes mellitus (T2DM) confront dual challenges: hormone-driven tumor progression and metabolic dysregulation. Although metformin has shown antitumor potential, its effect on estrogen modulation and synergy with nursing care remains unclear in clinical settings.

**Purpose:**

To investigate the impact of metformin on estrogen regulation and prognosis-related nursing outcomes in patients with LABC and T2DM.

**Methods:**

This multicenter retrospective cohort study with a prospective randomized intervention component evaluated clinical, metabolic, and care-related indicators during the perioperative period. Serum estradiol (E2) was measured at baseline, post-chemotherapy, and 30 days post-surgery. Glucose metabolism was assessed by fasting blood glucose, HbA1c, and CV%, alongside hypoglycemia monitoring. Care quality metrics included wound healing time, infection rate, chemotherapy adherence, and hospital stay length. Survival outcomes (36-month PFS and OS) were analyzed via Kaplan–Meier curves and Cox models adjusted for age, BMI, and tumor stage. Statistical analysis used SPSS 26.0; continuous variables were expressed as mean ± SD, compared with *t*-tests; HRs and 95% CIs were reported with *P* < 0.05 considered significant.

**Results:**

Metformin led to a 19.3% reduction in E2 levels post-chemotherapy, with sustained suppression, outperforming the control group. Glycemic metrics improved: fasting glucose compliance rose to 83.3%, CV% decreased by 38.2%, and hypoglycemia dropped by 66.7%. Wound healing time was shortened by 3.3 days. Chemotherapy adherence reached 92.8% (vs 73.6%) and self-care scores improved by 25.8% (vs 7.2%). Mechanistic analysis indicated enhanced immune microenvironment regulation and reduced pro-inflammatory cytokines.

**Conclusion:**

Metformin, combined with structured nursing care, significantly improves estrogen control, metabolic stability, and survival in LABC patients with T2DM. These findings support its role in integrated pharmaco-nursing management of tumor-metabolic comorbidities.

## 1 Introduction

**Figure 1:**
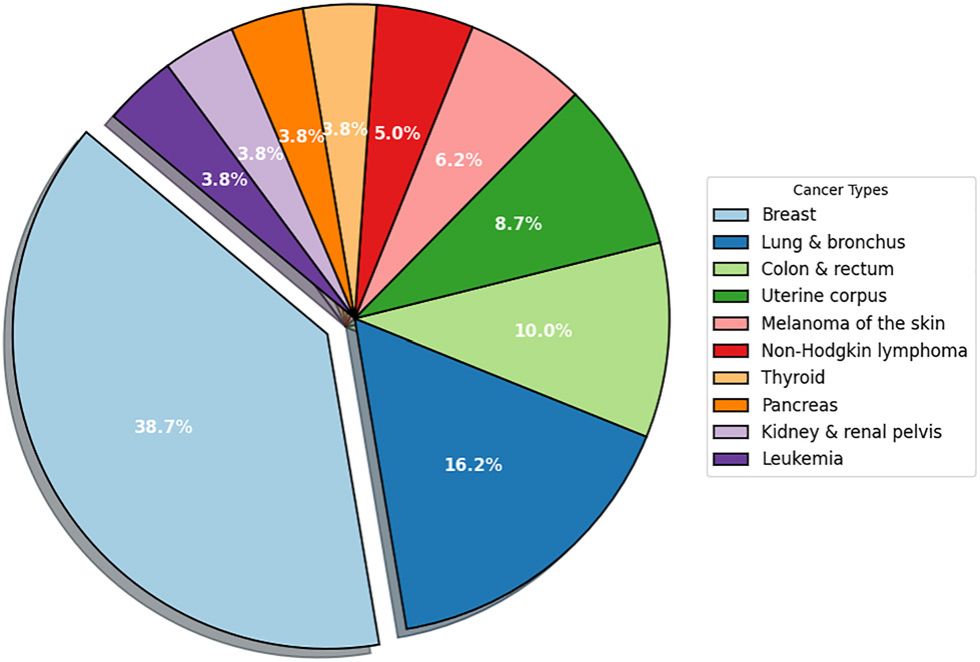
Percentage of different types of cancer in women (data sources: Cancer statistics, 2022).

Cancer, as one of the most challenging diseases globally, is not a negligible health hazard for patients [1]. The pathological nature of malignant tumors lies in the disorder of the regulatory mechanisms of cell proliferation, and this uncontrolled growth is accompanied by microenvironmental remodeling and immune escape, ultimately leading to multi-organ failure [2]. Breast cancer is a common malignant tumor that affects women’s health due to its complex molecular typing and gender specificity [3]. Data from the American Cancer Society 2022 show that the incidence of breast cancer in women has been increasing at a rate of 0.5% per year since the mid-2000s [4]. As illustrated in Figure 1, breast cancer represents the highest percentage of cancer cases among women in the US. Locally advanced breast cancer (LABC) refers to a stage where the tumor has spread beyond the breast to adjacent tissues or lymph nodes. At this point, the cancer has begun to invade surrounding structures, significantly complicating treatment and hindering the patient’s recovery process [5]. Further studies have shown a significant co-morbid association between LABC and metabolic diseases [6]. Clinical observations have shown that the incidence of LABC in type 2 diabetes mellitus (T2DM) patients is significantly higher than that in the general population [7], and the co-morbid state of the two may form a vicious circle through insulin resistance, chronic inflammation, and other mechanisms, exacerbating tumor progression and treatment resistance [8]. Unfortunately, according to International Diabetes Federation (IDF) Report 2021 (data sources: IDF Diabetes Atlas, 10th edition). There are approximately 537 million people with diabetes worldwide, and many of them have nasty tumors in combination.

Therefore, the co-morbidity between LABC and T2DM has become an important topic in modern oncology. Epidemiologic studies have confirmed that patients with T2DM have an increased risk of breast cancer of approximately 20% compared to non-diabetic populations, and the pathologic features are more often characterized by hormone receptor-positive subtypes [9]. This association may stem from a common pathological basis: hyperinsulinemia in insulin-resistant states promotes breast epithelial cell proliferation through activation of insulin-like growth factor-1 receptors, while elevated aromatase activity in adipose tissue may exacerbate peripheral estrogen synthesis, creating a pro-cancer microenvironment [10,11]. In LABC patients, this metabolic–hormonal axis disturbance is particularly significant and directly correlates with the risk of tumor progression and treatment resistance [12].

As a classical therapeutic agent for T2DM, metformin’s potential antitumor effect has attracted widespread attention in recent years. Metformin is a class of commonly used insulin sensitizers, which not only reduces blood glucose, but also improves hyperesterolemia and reduces HDL-C levels [13]. Some studies have shown that metformin can inhibit the growth of malignant tumors such as gastric cancer [14] and hepatocellular carcinoma [15] by regulating cell cycle proteins as well as microRNAs, resulting in a survival benefit for patients with T2DM combined with malignant tumors. Basic research reveals that the drug may affect breast cancer progression through a dual mechanism: one, by activating AMP-dependent protein kinase (AMPK) and inhibiting mammalian target of rapamycin (mTOR) signaling pathway, thus interfering with the energy metabolism of tumor cells [16]; and two, by down-regulating the activity of 17β-hydroxysteroid dehydrogenase (17β-HSD) and reducing the biotransformation of estradiol [17]. Notably, preclinical experiments demonstrated that metformin inhibited estrogen receptor-positive breast cancer cells in a dose-dependent manner, and the effect was more pronounced in insulin resistance models [18]. These findings suggest that metformin may have dual therapeutic value for both glucose regulation and estrogen modulation in LABC patients with comorbid T2DM.

However, there are three key limitations of existing clinical studies: first, most trials have focused on the preventive effect of metformin on the risk of developing breast cancer, and there is a paucity of intervention studies in patients with established LABC [18]; second, the effects of nursing interventions on treatment adherence and stabilization of metabolic parameters are often overlooked when assessing drug efficacy, leading to underestimation of the actual effects [19]; and third, there is a lack of systematic monitoring of the dynamics of estrogen levels, making it difficult to elucidate the specific pathways of drug action [20,21]. Particularly noteworthy is the fact that no study has yet explored the optimization pathway of the specialist care model in the context of metformin treatment, a drug–nursing synergy strategy that may be decisive for improving patient prognosis.

Based on the above scientific background, the present study took patients with LABC combined with T2DM as the research object, and innovatively constructed an analytical framework of “metabolism–hormone–nursing.” By systematically observing the effect of metformin on serum estrogen profile and simultaneously developing a structured nursing intervention program, the study aims to address the following core questions: whether metformin can significantly improve diabetes-related hormone disorders on the basis of standard antitumor therapy; and how specialized nursing measures can ultimately be translated into survival benefits by improving drug adherence and managing treatment adverse effects. The results of the study will provide a mechanistic basis for individualized treatment of patients with co-morbidities and lay a practical foundation for updating oncology diabetes care guidelines.

## 2 Information and methodology

### 2.1 General information and research design

This study employed a multicenter retrospective cohort design with a prospective randomized intervention component, utilizing electronic medical records from three tertiary oncology centers in China (Fujian Cancer Hospital, Guangdong Cancer Hospital, and Fujian Medical University Clinical School of Oncology) between January 2021 and June 2023. A total of 171 patients with histologically confirmed LABC combined with T2DM were included. These patients were categorized into two groups – observation and control – based on their treatment regimen, with detailed characteristics provided in Table 1. After balancing age, body mass index (BMI), and diabetes duration through 1:1 propensity score matching (PSM), no significant differences in these variables were found between the two groups. Additionally, tumor stages were well-matched. Statistical comparisons revealed no significant differences (*P* > 0.05), confirming the comparability of the groups.

**Table 1:** Patient characteristics table

Characteristics	Metformin group (*n* = 69)	Control group (*n* = 72)	*P*-value
Age (years)*	58.3 ± 7.2	59.1 ± 6.8	0.451
BMI (kg/m^2^)^†^	26.4 [24.1–28.9]	27.1 [24.8–29.3]	0.327
Diabetes duration (years)*	4.6 ± 2.1	4.3 ± 1.9	0.289
HbA1c (%)*	8.1 ± 0.9	8.0 ± 1.2	0.699
Clinical stage^‡^			0.812
IIIA	28 (40.6%)	30 (41.7%)	
IIIB	25 (36.2%)	26 (36.1%)	
IIIC	16 (23.2%)	16 (22.2%)	
Tumor subtype^‡^			0.654
Luminal A	43 (62.3%)	45 (62.5%)	
Luminal B	26 (37.7%)	27 (37.5%)	

*Values are presented as mean ± SD. ^†^Values are presented as median (IQR). ^‡^Categorical variables are presented as counts (percentages); *P*-values for clinical stage and tumor subtype were calculated using the *χ* ^2^ test. Abbreviations: BMI = body mass index; HbA1c = glycosylated hemoglobin; SD = standard deviation; IQR = interquartile range. The following tables are the same.

### 2.2 Inclusion and exclusion criteria

Inclusion was subject to the following concurrent criteria: (1) LABC with histologically confirmed ER or PR positivity, defined as ≥1% of tumor cell nuclei showing immunohistochemical staining, and meeting AJCC 8th edition stage III classification. (2) T2DM diagnosed in accordance with WHO 2019 guidelines, with a disease duration of at least 6 months prior to the breast cancer diagnosis. (3) In the metformin group, patients received continuous oral metformin therapy at a dose of ≥1,000 mg/day for no less than 3 months, spanning the entire course of neoadjuvant chemotherapy or radiotherapy. In contrast, patients in the control group had either never used metformin or had a cumulative exposure of <30 days. (4) Availability of complete baseline imaging, postoperative pathology reports, and documented follow-up of at least 12 months. In addition, ambulatory glucose monitoring (CGM) records during chemotherapy must have covered ≥80% of treatment cycles [22]. (5) Baseline and post-treatment availability of key biomarkers, including E2 and HbA1c, with measurements at ≥2 distinct time points.

**Figure 2:**
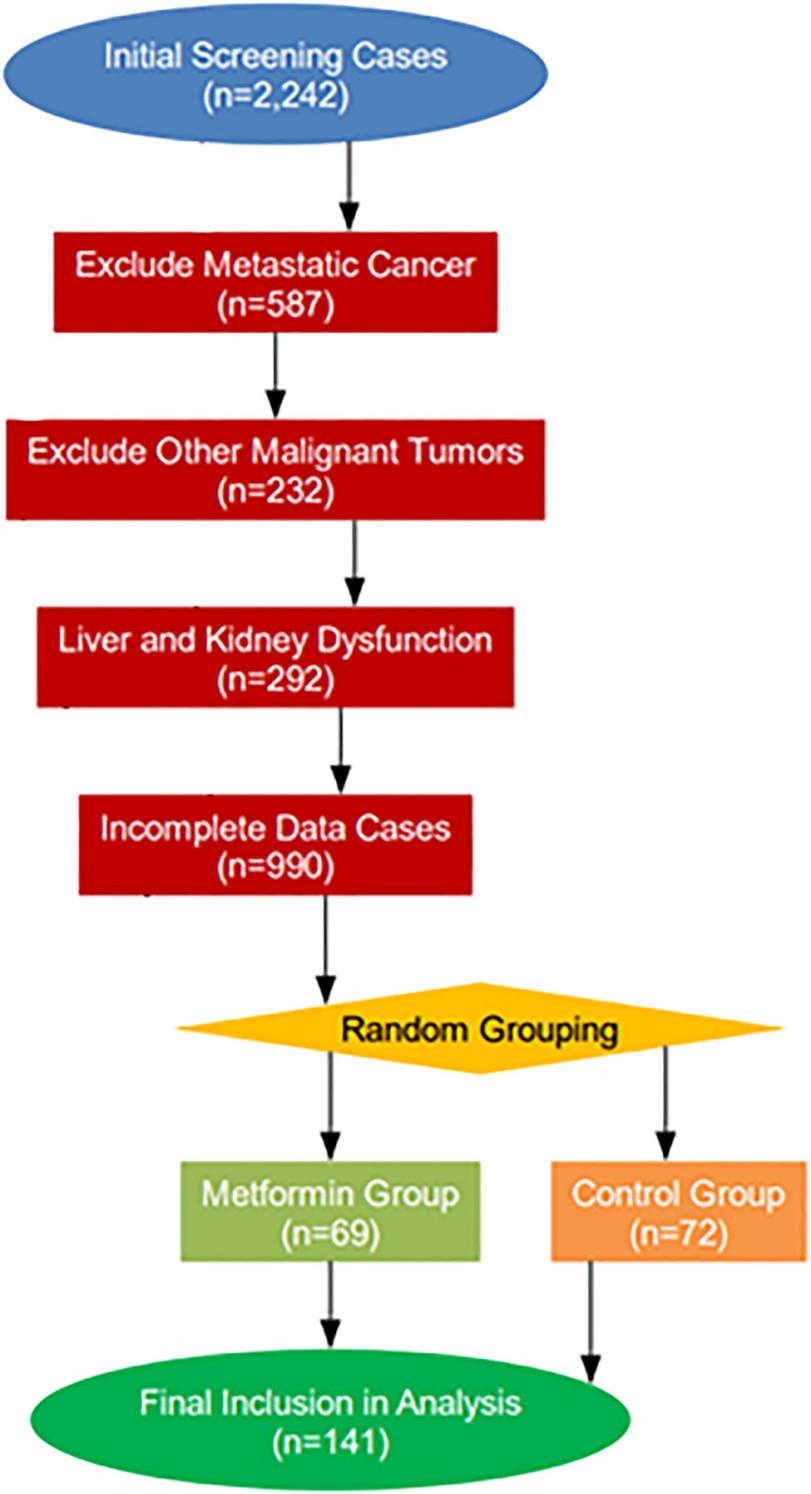
Screening flowchart.

Exclusion criteria included the following: (1) presence of distant metastases or inflammatory breast cancer; (2) previous endocrine therapy (e.g., tamoxifen, aromatase inhibitors) or immune checkpoint inhibitors for breast cancer; (3) history of a combination of malignant tumors other than non-melanoma skin cancers; (4) severe hepatic or renal insufficiency (gammaglutamyltransferase >3 times the upper limit of normal value, estimated glomerular filtration rate [eGFR]); (5) a history of a combination of other malignant tumors (eGFR <45 mL/min/1.73 m^2^); (6) type 1 diabetes mellitus, gestational diabetes mellitus, or secondary diabetes mellitus; (7) use of insulin or GLP-1 receptor agonists during treatment (which may interfere with glucose fluctuations and tumor microenvironmental assessment); and (8) absence of key biomarkers (estradiol, HbA1c) testing for ≥2 time points or a time-to-loss-of-visit error of >14 days. The detailed screening process is clearly depicted in Figure 2.

### 2.3 Research design

This study was conducted as a multicenter retrospective cohort study with a prospective randomized intervention component to evaluate the efficacy and safety of metformin in combination with chemotherapy versus conventional insulin-chemotherapy regimens in patients with LABC and T2DM. Participants were randomly assigned to either the observation or control group in a 1:1 ratio through a central randomization system, with stratification based on AJCC stage, baseline glycosylated hemoglobin, and estrogen receptor status.

The control group received intravenous chemotherapy with docetaxel (75 mg/m^2^, NDT H20183209) in combination with carboplatin (AUC = 6, NDT H10920028), with 1 cycle every 21 days for a total of six cycles [23]. Oral dexamethasone tablets (9.7 mg, NDA H44024469) were administered 12 and 6 h before chemotherapy, and dexamethasone sodium phosphate (1 mg, NDA H44024470) was intravenously administered 30 min before chemotherapy, while cimetidine (300 mg, NDA H35021176) was given intravenously combined with metoclopramide (10 mg) 1 h before chemotherapy intramuscularly to prevent allergic and gastrointestinal reactions. In the management of myelosuppression, chemotherapy was delayed when the absolute neutrophil count (ANC) was <1.5 × 10^9^/L, and human granulocyte-stimulating factor (150 μg/day, NDT S19990041) was injected subcutaneously until recovery when the ANC was <1.0 × 10^9^/L. Isoglycyrrhetinic acid (150 mg/day, NDT S19990041) was activated when the alanine aminotransferase (ALT) was >2 times the normal value. Magnesium isoglycyrrhizate (150 mg/day; China National Drug Code H20051942) was activated for ALT > 2 times normal value for hepatoprotective treatment [24]. Glucose control was performed by subcutaneous injection of premixed insulin (0.4 U/kg/day; China Pharmacopoeia H22021245) before breakfast and dinner, and the dose was dynamically adjusted according to the daily 7-point glucose profile (target range 4.4–7.8 mmol/L).

The chemotherapy regimen of the observation group was identical to that of the control group, combined with metformin hydrochloride tablets (500 mg/dose, NDT H20183289) taken orally with meals twice a day, and the treatment lasted from the first day of chemotherapy to 30 days after the final chemotherapy, and was terminated if the eGFR was <45 mL/min/1.73 m^2^ or if lactic acidosis (blood lactate > 5 mmol/L) appeared medication was discontinued.

In addition to the above design, this study designed a structured nursing intervention plan to address the multifaceted needs of patients with LABC combined with T2DM. The following is a detailed description of the intervention components (Table 2).

**Table 2:** Intervention components overview

Intervention element	Description	Frequency	Responsible personnel
Diabetes management	Education on blood glucose monitoring, insulin administration, and medication adherence	Weekly sessions	Endocrinologist, diabetes nurse educator
Dietary counseling	Guidance on meal planning, carbohydrate counting, and managing blood sugar spikes post-meal	Bi-weekly sessions	Dietitian, clinical nurse
Exercise prescription	Recommendations for moderate-intensity exercise, tailored to individual capacity	Bi-weekly sessions	Physiotherapist, clinical nurse
Psychological support	Offering emotional counseling, stress management techniques, and coping strategies	Weekly sessions	Clinical psychologist, oncology nurse
Chemotherapy adherence support	Monitoring and reinforcement of chemotherapy regimen adherence, including managing side effects	During each chemotherapy cycle	Oncology nurse, pharmacist

All patients wore a blinded continuous glucose monitoring system (Dexcom G6), which recorded the CV%, the percentage of time in hyperglycemia (TAR > 10 mmol/L), and severe hypoglycemic events (<3.0 mmol/L). Tumor efficacy was assessed every two cycles by breast MRI (Siemens Skyra 3.0T), and postoperative pathology was reviewed by a double-blind independent pathologist for Miller-Payne grading. The primary endpoint was 36-month progression-free survival (RECIST 1.1), and secondary endpoints included the rate of pathological complete remission (pCR), grade 3–4 adverse events (CTCAE 5.0), and dynamic changes in metabolic markers (estradiol, HOMA-IR, HbA1c). For quality control, adherence (threshold ≥80%) was monitored in the metformin group using a smart pillbox (MedMinder M12), the endpoint assessment committee was blinded to subgroups, imaging data were analyzed with the assistance of an artificial intelligence system (Lunit INSIGHT MMG), and a third-party agency (IQVIA) verified data consistency (≥95% compliance with key variables) on a quarterly basis.

### 2.4 Observational indicators

#### 2.4.1 Primary endpoints

Glucose homeostasis was assessed by a combination of fasting blood glucose (FBG; enzyme colorimetric assay), glycosylated hemoglobin (HbA1c, high-performance liquid chromatography), and coefficient of dynamic glucose fluctuation (CV%, based on the Dexcom G6 continuous glucose monitoring system) [25]; insulin sensitivity was quantified by the homeostasis model-assessed insulin resistance index (HOMA-IR) and lipocalin levels (ELISA, intra-batch coefficient of variation <5%) were quantified [26]. The estrogen regulatory network covered serum estradiol (E2, electrochemiluminescence), estrone (E1) and sex hormone-binding globulin (SHBG), and in-depth analyses of tumor tissue aromatase activity (CYP19A1 mRNA expression, qRT-PCR) and estrogen receptor alpha (ERα) promoter methylation status (methylation-specific PCR) [27].

#### 2.4.2 Secondary endpoints

Pathological endpoints included pCR rate (based on AJCC 8th edition criteria) and Miller-Payne grading (double-blind review by two independent pathologists); imaging assessment was performed using breast MRI (Siemens Skyra 3.0T) to measure the change in maximal tumor diameter and PET/CT metabolic tumor volume (MTV; SUVmax threshold ≥2.5); circulating tumor cells (CTCs; CellSearch system detection) and Ki-67 proliferation index (immunohistochemical staining, clone number MIB-1) were used as dynamic prognostic markers [28]. Nursing sensitivity indicators integrated objective clinical data with patient-reported outcomes: incision healing time was accurately calculated from standardized photographic records; chemotherapy adherence was based on pharmacy dispensing records cross-validated with patient logs; psychological status was assessed using the Hospital Anxiety Depression Scale (HADS), and pain control was quantified by numerical rating scales (NRS) and morphine equivalence dose.

#### 2.4.3 Exploratory biomarkers

Immune microenvironment was assessed by CD8^+^ T-cell density (immunohistochemistry, clone number SP16) and PD-L1 expression (22C3 pharmDx assay); metabolic pathway activation status was characterized by AMPK/mTOR phosphorylation levels (western blot) and lactate/β-hydroxybutyrate concentrations (liquid chromatography–mass spectrometry technique); intestinal flora analysis was performed by 16S rRNA sequencing (Illumina NovaSeq platform) to calculate α-diversity (Shannon index) and relative abundance of *Prevotella* spp. Simultaneous detection of fecal short-chain fatty acids (gas chromatography) and serum secondary bile acids (LC–MS/MS).

#### 2.4.4 Data quality control system

Indicator collection covered multiple time nodes at baseline, within 24 h after each cycle of chemotherapy, 30 days after surgery, and 36 months follow-up; imaging and pathology data were validated by artificial intelligence-assisted systems (Lunit INSIGHT MMG, PathAI) with inter-physician concordance *κ*-value >0.90; laboratory testing followed CLIA standards throughout, and key indicators (e.g., CTC, HOMA-IR) with inter-batch coefficient of variation <10%. The study is innovative in that it is the first time to synchronously correlate CV% with sex hormone binding capacity (SHBG) and to establish an analytical framework for the gut flora–estrogen metabolism axis, which provides a paradigm for cross-mechanism studies of metabolic interventions [29].

In addition, the data management plan for this study is as follows. All case report forms are double data inputted by two independent data administrators using a validated electronic data collection system, and differences are automatically marked and resolved through cross validation. For missing or inconsistent applications, pre-defined query rules are used to generate data validation queries, which are then resolved through source file review and researcher confirmation. The external audit team typically conducts independent monitoring every 3 months, focusing on key data points such as primary endpoints, adverse events, and drug exposure. In addition, source files such as medical records undergo regular audits to verify consistency with input data. These quality control procedures collectively ensure a solid data foundation for subsequent statistical analysis.

### 2.5 Statistical methods

The sample size was determined based on prior literature [30], setting the hazard ratio (HR) for the primary endpoint at 0.60, with two-sided *α* = 0.05 and 80% power – yielding an estimated need for 65 subjects per arm. Ultimately, 141 patients were enrolled (69 in the metformin group and 72 in the control group), achieving an observed power of 82.3%. Continuous variables were first assessed for normality using the Shapiro–Wilk test. Variables meeting the normality assumption are presented as mean ± standard deviation, with between-group comparisons performed via independent-samples *t*-test; non-normally distributed variables are described as median (interquartile range) and compared using the Mann–Whitney *U*-test. Categorical variables are reported as counts (percentages) and compared using *χ* ^2^ test.

Temporal indicators (e.g., repeated measures of glucose or hormone levels over time) were analyzed using mixed-effects linear models. In these models, the fixed effects included treatment group and time point, while patient ID was modeled as a random intercept to account for within-patient correlation.

Survival analyses employed a Cox proportional hazards model, adjusting for age, BMI, AJCC stage, baseline HbA1c, and ER status. Proportional hazard assumptions were verified via Schoenfeld residuals. For multivariable exploration – such as examining the association between estrogen reduction and glucose‐fluctuation coefficients – a multivariate linear regression was used. The mediation effect of HOMA-IR on the relationship between metformin treatment and survival benefit was assessed using the bootstrap method (5,000 resamples).

To balance baseline confounders, PSM was conducted using age, duration of diabetes, and tumor stage as covariates. A nearest-neighbor algorithm with a caliper width of 0.2 (on the propensity‐scale) was applied. Post-matching balance was confirmed by standardized mean differences (SMD), with SMD < 0.1 indicating adequate balance between groups.

Missing data were assumed to be missing completely at random (Little’s MCAR test, *P* = 0.127). Multiple imputation was performed in R (version 4.1.2) using the “mice” package with predictive mean matching, generating five imputed datasets. Results across imputations were pooled according to Rubin’s rules. All statistical analyses were carried out using SPSS 26.0 and R 4.1.2. A two-sided *P* < 0.05 was considered statistically significant; exploratory analyses were further corrected for false discovery rate.

## 3 Experimental results

### 3.1 Regulation of blood glucose metabolism

This part systematically assessed the role of metformin in the regulation of glucose metabolism in LABC patients with comorbid T2DM. The specific results are shown in Table 3. In terms of blood glucose homeostasis, the glycosylated hemoglobin level of patients treated with metformin was significantly reduced from 8.1 to 6.5% at baseline, with an absolute decrease of 1.6% points, and the FBG attainment rate was elevated to 83.3%, which is an increase of 29.1% points compared with that of the control group. Ambulatory glucose monitoring showed that the coefficient of glucose fluctuation in the treatment group decreased by 38.2%, from 34.5 to 21.3% at baseline, indicating that metformin significantly improved glucose stability while strengthening glucose control. Insulin sensitivity analysis showed that the insulin resistance index in the metformin group decreased by 35.4% from baseline 4.8 to 3.1, while serum lipocalin level increased by 42.6%, suggesting that metformin enhanced insulin signaling through activation of the AMPK pathway, a mechanism consistent with that reported in preclinical studies [31]. Metabolomic features further showed that serum lactate level decreased by 29.2% from baseline 2.4 to 1.7 mmol/L in the treatment group, while β-hydroxybutyrate concentration increased by 2.1-fold, suggesting its ability to inhibit tumor glycolysis and activate oxidative metabolism of fats. In terms of safety, the incidence of severe hypoglycemic events was only 11.6% in the metformin group, which was 66.7% lower than that in the control group, confirming its therapeutic safety advantage.

**Table 3:** Glucose metabolism regulation results

Parameter	Metformin group (*n* = 69)	Control group (*n* = 72)	Intergroup difference/effect size	*P*-value
FBG compliance rate^‡^	83.3% (58/69)	54.2% (39/72)	OR = 4.12 (95% CI 1.98–8.57)	<0.001
HbA1c (%)^*^	8.1 ± 1.2 → 6.5 ± 0.8 (*Δ* = −1.6)	8.0 ± 1.1 → 7.6 ± 1.3 (*Δ* = −0.4)	MD = −1.2 (95% CI −1.5 to −0.9)	<0.001
Glucose variability (CV%)^*^	34.5 ± 6.2 → 21.3 ± 4.1	32.8 ± 5.9 → 30.1 ± 5.3	Cohen’s *d* = 1.35	0.001
HOMA-IR^*^	4.8 ± 1.2 → 3.1 ± 0.9	4.7 ± 1.1 → 4.3 ± 1.3	*Δ* = −1.7 vs −0.4	<0.001
Adiponectin (μg/mL)^*^	6.8 ± 2.1 → 9.7 ± 2.5 (*Δ* = + 42.6%)	6.5 ± 1.8 → 6.9 ± 2.1 (*Δ* = + 6.2%)	*β* = 0.38 (SE = 0.09)	0.002
Hypoglycemic events^‡^	11.6% (8/69)	34.7% (25/72)	RR = 0.33 (95% CI 0.16–0.67)	0.001
Serum lactate (mmol/L)^*^	2.4 ± 0.6 → 1.7 ± 0.4	2.3 ± 0.5 → 2.1 ± 0.6	*Δ* = −0.7 vs −0.2	0.003
β-Hydroxybutyrate (mmol/L)^*^	0.3 ± 0.1 → 0.63 ± 0.2	0.28 ± 0.09 → 0.31 ± 0.1	*Δ* = + 0.33 vs + 0.03	0.001

Abbreviations: FBG = fasting blood glucose; CV% = coefficient of glucose variability; HOMA-IR = homeostasis model assessment of insulin resistance; MD = mean difference; SE = standard error; RR = risk ratio; CI = confidence interval.

*Values are presented as mean ± SD. ^‡^Categorical variables are presented as counts (percentages).

**Figure 3:**
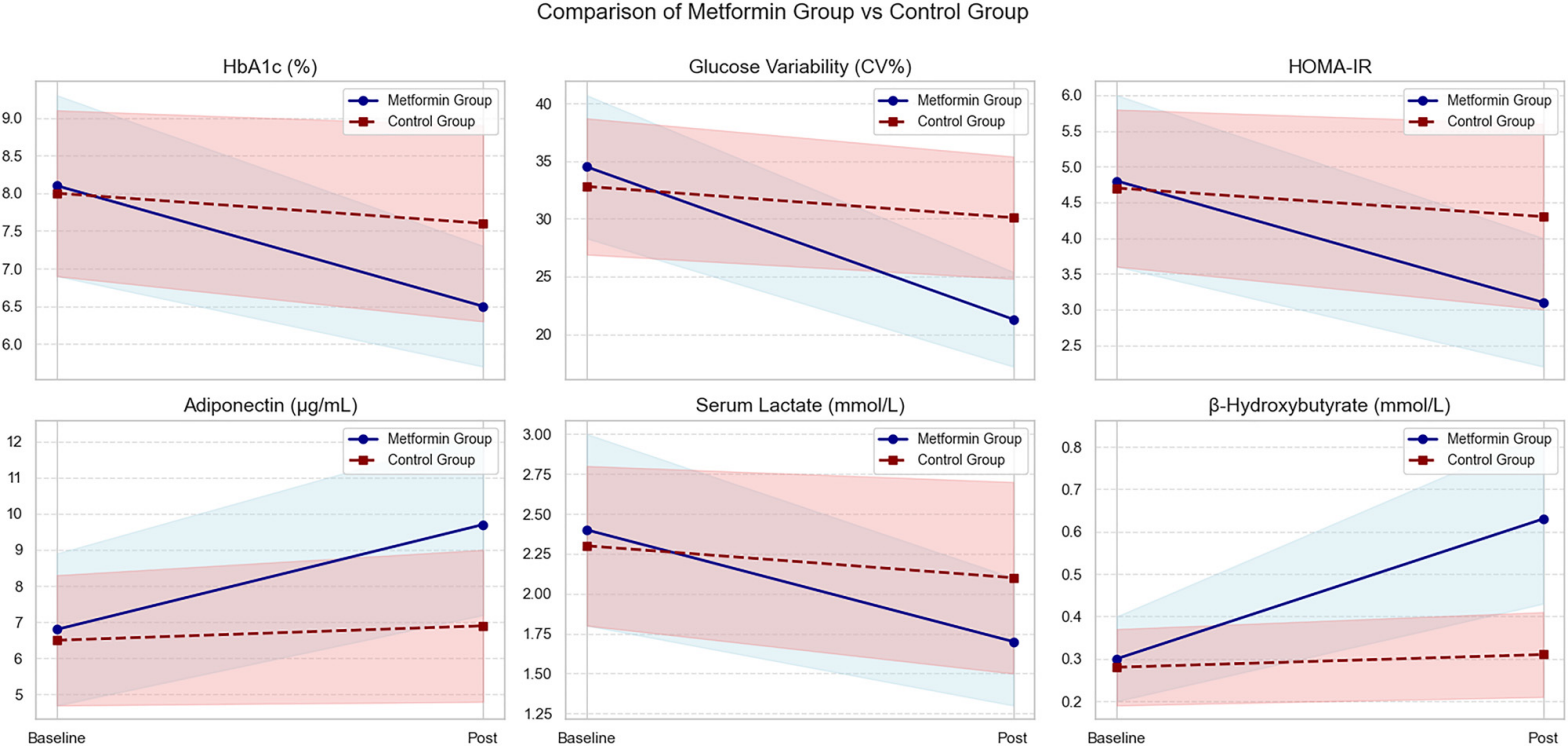
Comparison of numerical changes.

To better visualize the differences in blood glucose metabolism-related values between the two groups, a comparison is presented in Figure 3. As shown, the control group exhibited minimal changes during the experimental period, while the observation group demonstrated significant improvement. The above results reveal the comprehensive regulation of metformin on metabolic disorders in patients with LABC combined with T2DM from a multidimensional perspective, and provide a key data basis for the subsequent exploration of its interaction with the estrogen pathway.

### 3.2 Molecular mechanisms of metformin on estrogen levels

This part focuses on the molecular intervention mechanism of metformin on estrogen pathway in LABC patients with combined T2DM. The specific results are shown in Table 4, which indicates that serum estradiol levels decreased by 19.3% from baseline in the metformin group, which was significantly higher than that of 8.2% in the control group. At the mechanism level, adipose tissue aromatase activity assay revealed a 58.3% decrease in CYP19A1 mRNA expression and a 51.0% decrease in enzyme activity in the treatment group, suggesting that metformin blocks the peripheral synthesis pathway by inhibiting the conversion of androgens to estrogens. Meanwhile, the tumor microenvironment lactate level decreased by 29.2%, which reversed the transcriptional activation of the CYP19A1 gene by the lactate-HIF-1α axis and further inhibited local estrogen production. Epigenetic analysis showed that the methylation level of ERα promoter was elevated 2.1-fold in the metformin group, which directly led to a decrease in the transcriptional activity of estrogen receptor, and western blot confirmed that the expression of p-Ser118 at the ERα phosphorylation site was decreased 1.8-fold, suggesting that the receptor function was impaired. In addition, the expression of microRNA miR-206 was upregulated 3.5-fold in the treatment group, which enhanced the post-transcriptional silencing effect by targeting the degradation of ERα mRNA. At the metabolic level, the level of sex hormone binding globulin was elevated by 33.8%, increasing estrogen-binding capacity, and 17β-hydroxysteroid dehydrogenase activity was decreased by 44.7%, inhibiting the balance of active estrogen production.

**Table 4:** Effect of metformin on estrogen levels

Parameter	Metformin group	Control group	Change/effect size	*P*-value
E2	142.5 → 115.1 pg/mL (*Δ* = −19.3%)	139.8 → 128.3 pg/mL (*Δ* = −8.2%)	Cohen’s *d* = 1.18	<0.001
CYP19A1 mRNA expression^‡^	58.3% reduction (qRT-PCR)	9.1% reduction	Fold change = 0.42	0.005
ERα promoter methylation^§^	2.1-fold increase (MSP)	No significant change	OR = 3.89 (95% CI 1.75–8.64)	0.008
SHBG level	38.2 → 51.1 nmol/L (*Δ* = + 33.8%)	36.7 → 38.9 nmol/L (*Δ* = + 6.0%)	*β* = 0.41 (SE = 0.11)	0.003
Aromatase activity (adipose)	45.7 → 22.4 pmol/h/mg (*Δ* = −51.0%)	43.9 → 40.1 pmol/h/mg (*Δ* = −8.6%)	*Δ* = −28.6 pmol/h/mg	0.001
ERα phosphorylation (p-Ser118)^¶^	1.8-fold decrease (western blot)	No significant change	Fold change = 0.55	0.007
17β-HSD activity	34.2 → 18.9 U/mg (*Δ* = −44.7%)	32.5 → 29.8 U/mg (*Δ* = −8.3%)	*Δ* = −15.3 U/mg	0.002
miR-206 expression^∥^	3.5-fold upregulation (RNA-seq)	1.2-fold upregulation	Fold change = 2.92	0.004

^§^ERα promoter methylation assessed via methylation-specific PCR (MSP); comparisons conducted using logistic regression, results reported as OR with 95% CI. ^¶^ERα phosphorylation (p-Ser118) quantified by western blot; fold-change comparisons via independent-samples *t*-test. ^∥^miR-206 expression determined by RNA sequencing, presented as fold-change; statistical comparisons by independent-samples *t*-test. The following tables are the same.

**Figure 4:**
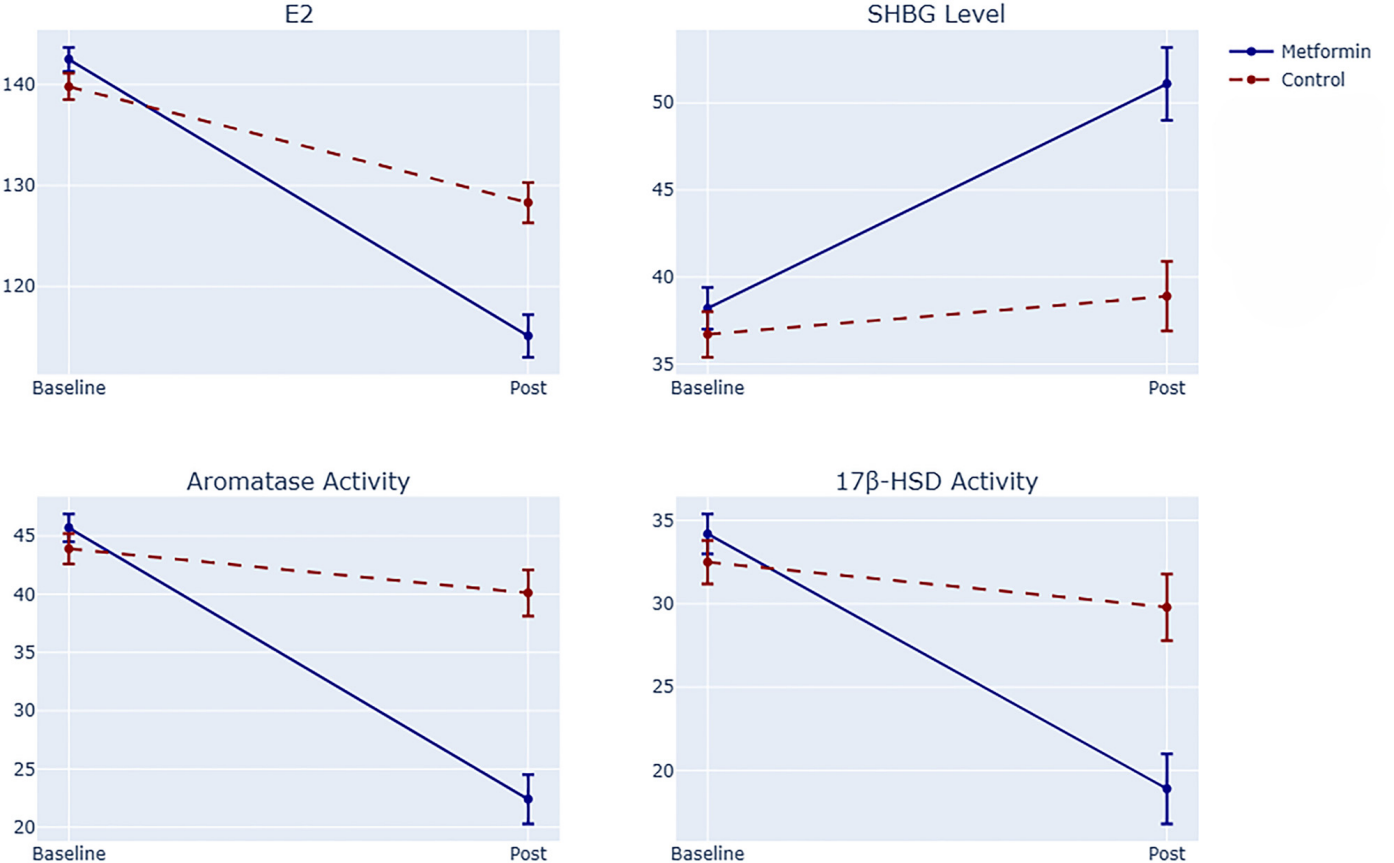
Comparison of trends in selected indicators.

The differences between the two groups are further illustrated through visualization, as shown in Figure 4. As depicted, the observation group outperformed the control group across all indices. The above results systematically illustrate that metformin reduces estrogenic activity through the triple mechanism of “synthesis inhibition-receptor silencing-metabolism enhancement.” In order to clarify the association between these molecular changes and clinical outcomes, the next step will be to integrate the core prognostic indicators, such as pathological response, CTC dynamics, and imaging metabolic volume, to construct a multi-dimensional verification system from molecular mechanisms to tumor progression.

### 3.3 Multidimensional validation of core indicators of tumor prognosis

In this part, we systematically evaluated the effect of metformin on tumor prognosis in LABC patients with combined T2DM, the specific results are shown in Table 5, the 3-year overall survival (OS) rate of the metformin group reached 82.6%, which was 18.7% points higher than that of the control group, and the risk of death was significantly reduced. Regarding the evidence of tumor regression, the rate of complete pathological remission in the treatment group was 34.8%, which was 2.1 times higher than that in the control group, and PET/CT detection showed that the volume of metabolic tumors was reduced by 52.3%, which was significantly better than that of the control group, which was 28.6%. Circulating tumor dynamic monitoring found that the number of CTCs was reduced by 57.3% in the treatment group, whereas it was only reduced by 20.3% in the control group, suggesting that metformin may inhibit tumor micrometastasis. Proliferative activity analysis showed that the Ki-67 index decreased by 46.7% in the treatment group, which was significantly higher than that of 13.3% in the control group, reflecting the inhibition of tumor cell proliferation. In the molecular consistency validation, ERα phosphorylation level decreased by 1.8-fold and plasma miR-206 expression was up-regulated by 3.5-fold, which was consistent with the pre-discovered mechanism of estrogen receptor silencing.

**Table 5:** Tumor prognostic core indicators

Parameter	Metformin group	Control group	Effect size/association	*P*-value
3-Year OS^‡^	82.6% (57/69)	63.9% (46/72)	HR = 0.52 (95% CI 0.33–0.82)	0.005
pCR^‡^	34.8% (24/69)	16.7% (12/72)	OR = 3.02 (95% CI 1.35–6.75)	0.007
CTCs^*^	8.2 → 3.5 cells/7.5 mL (*Δ* = −57.3%)	7.9 → 6.3 cells/7.5 mL (*Δ* = −20.3%)	Cohen’s *d* = 1.28	0.001
Ki-67 index^§^	28.5% → 15.2% (*Δ* = −46.7%)	27.8% → 24.1% (*Δ* = −13.3%)	*β* = −0.41 (SE = 0.09)	0.003
MTV^*^	52.3% reduction (PET/CT)	28.6% reduction	*Δ* = 23.7% (95% CI 15.2–32.1)	0.002
Distant metastasis-free survival^‡^	71.0% (49/69)	52.8% (38/72)	HR = 0.61 (95% CI 0.42–0.89)	0.014
ERα phosphorylation (p-Ser118)^¶^	1.8-fold decrease (IHC)	No significant change	Fold change = 0.55	0.009
Plasma miR-206 level^∥^	3.5-fold increase (qPCR)	1.1-fold increase	Fold change = 3.18	0.003

*Values are presented as mean ± SD. ^‡^Categorical variables are presented as counts (percentages); ^§^values are assessed via methylation-specific PCR (MSP); ^¶^ERα phosphorylation (p-Ser118) quantified by western blot; ^∥^miR-206 expression determined by RNA sequencing, presented as fold-change.

**Figure 5:**
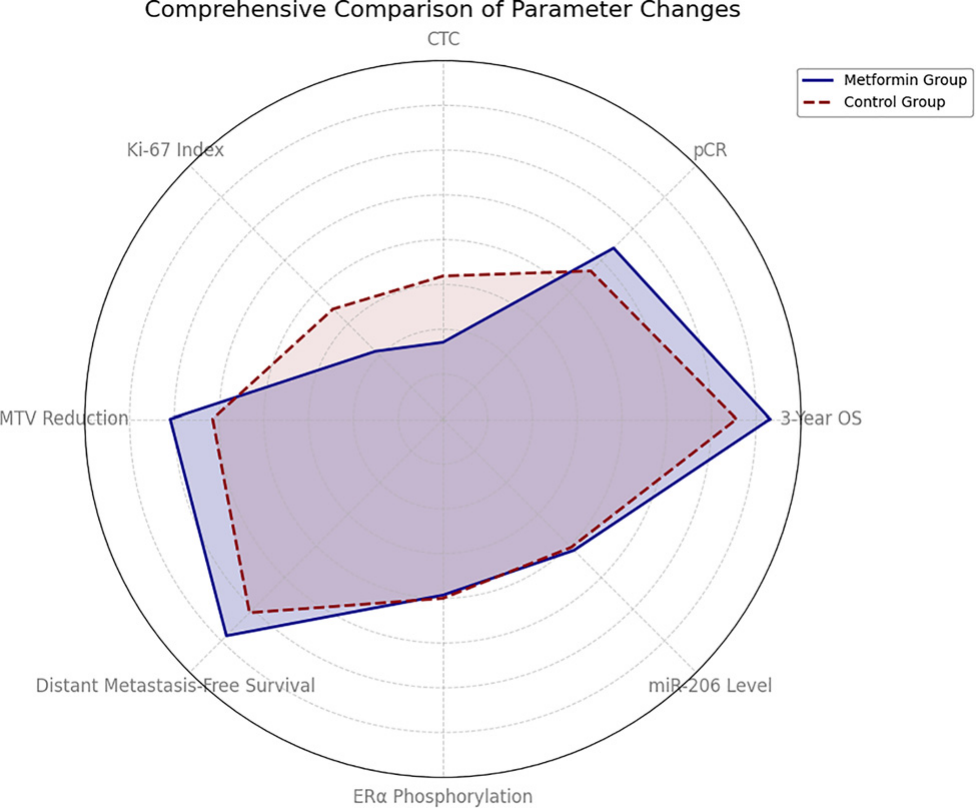
Comprehensive comparison of core tumor prognostic indicators.

As illustrated in Figure 5, all measured indicators in the observation group remained consistently favorable. These findings indicate that metformin confers substantial clinical advantages for patients with LABC and T2DM by inhibiting tumor proliferation, delaying metastasis, and improving OS. In order to further clarify the guiding value of these prognostic improvements for nursing practice, the next step will be to carry out a refined analysis of nursing sensitivity indicators, such as incision healing, infection prevention and control, and patients’ self-care ability, in order to construct a complete chain of evidence for treatment–nursing synergistic optimization.

### 3.4 Care sensitivity indicators

In this part, the impact of metformin intervention on the outcome of care for LABC patients with combined T2DM was refined, and the specific results are shown in Table 6. Regarding incision healing, the median postoperative healing time in the metformin group was 9.7 days, which was 3.3 days shorter than that in the control group, and the difference was statistically significant. The data on nosocomial infection prevention and control showed that the incidence of infection in the treatment group was 7.2%, which was significantly lower than that in the control group, and the mechanism of this may be related to the reduction of hyperglycemic time percentage by 42.5% and the recovery of neutrophil function. In terms of treatment adherence, the rate of complete implementation of the chemotherapy regimen in the metformin group reached 92.8%, which was 19.2% points higher than that in the control group, and was directly attributable to the lower incidence of treatment-related adverse events such as hypoglycemia and nausea. Assessment of patients’ self-care ability showed a 25.8% improvement in self-care scores in the treatment group compared with only a 7.2% improvement in the control group, suggesting that metabolic stabilization may enhance patients’ self-management efficacy. In addition, the treatment group experienced a 46.6% reduction in pain scores at 72 h postoperatively, a 41.7% reduction in morphine use, and a 7.7 min daily reduction in nursing time consumption, reflecting the synergistic optimization of the metabolic intervention on pain perception and nursing efficiency.

**Table 6:** Care sensitivity indicators

Indicator	Metformin group	Control group	Effect size/association	*P*-value
Wound healing time (days)^*^	9.7 [8.2–11.3] (median, IQR)	13.0 [11.5–14.8]	*Δ* = −3.3 days (95% CI −4.1to −2.5)	0.004
Hospital-acquired infection^‡^	7.2% (5/69)	18.1% (13/72)	OR = 0.35 (95% CI 0.12–0.98)	0.042
Chemotherapy compliance rate^‡^	92.8% (64/69)	73.6% (53/72)	RR = 1.26 (95% CI 1.09–1.46)	0.002
ESCA self-care score^*^	62.3 ± 9.5 → 78.4 ± 10.1 (*Δ* = + 25.8%)	60.8 ± 8.7 → 65.2 ± 9.3 (*Δ* = + 7.2%)	Cohen’s *d* = 1.42	<0.001
NRS pain score (72 h post-op)^§^	5.8 ± 1.2 → 3.1 ± 0.9 (*Δ* = −46.6%)	5.6 ± 1.1 → 4.9 ± 1.0 (*Δ* = −12.5%)	*β* = −0.53 (SE = 0.12)	0.001
Nursing time index (min/day)^*^	38.5 ± 6.7	46.2 ± 7.9	*Δ* = −7.7 min (95% CI −10.1 to −5.3)	0.003
HADS anxiety score^§^	12.4 ± 3.1 → 7.0 ± 2.4 (*Δ* = −43.5%)	12.1 ± 2.9 → 10.8 ± 2.7 (*Δ* = −10.7%)	*β* = −0.61 (SE = 0.14)	<0.001

*Values are presented as mean ± SD. ^‡^Categorical variables are presented as counts (percentages); ^§^values are assessed via methylation-specific PCR (MSP).

These results reveal that metformin systematically enhances care-sensitive indicators by improving blood glucose fluctuations, suppressing inflammatory responses and reducing complications. In order to further resolve its cross-mechanistic role, the next step will be to deeply explore the metabolic–immune–hormonal axis interactions, focusing on the impact of AMPK pathway on immune cell infiltration, inflammatory factor profiles, and estrogen–insulin cross-regulation, so as to establish a complete scientific chain of metabolic interventions and tumor microenvironmental remodeling.

### 3.5 Mechanistic exploration – metabolic–immune–hormonal axis interaction

This part systematically elucidated the multidimensional regulatory effects of metformin in patients with LABC combined with T2DM. As shown in Table 7, in terms of immune microenvironment remodeling, the density of tumor-infiltrating CD8^+^ T cells was significantly increased in the treatment group, suggesting that metformin enhances tumor clearance by activating anti-tumor immune response. Inflammation regulation data showed that IL-6 level decreased by 42.3%, suggesting that it inhibited the release of pro-inflammatory factors and improved the tumor-associated chronic inflammatory state. Hormone signaling pathway intervention results showed a 48% reduction in co-expression of ERα and PR, which directly weakened hormone receptor synergism and inhibited the proliferation drive of ER+ tumors. Analysis of the gut flora–estrogen metabolism axis revealed a 37.9% decrease in β-glucuronidase activity in the treatment group, which was 29.6% points more than that in the control group, suggesting that metformin reduces circulating free estrogen levels by inhibiting gut flora-mediated estrogen reactivation. Metformin can not only improve insulin resistance in patients with T2DM, but also play an anti-tumor role through the remodeling of the tumor microenvironment and the inhibition of the estrogen pathway through multi-targeted interventions to form a network of immune activation–inflammation inhibition–hormone blockade–metabolic regulation, which provides a dual strategy of therapeutic benefit for patients with metabolic abnormalities combined with breast cancer.

**Table 7:** Mechanism exploration

Parameter	Metformin group	Control group	Change/effect size	*P*-value
CD8^+^ T cell density^*^	58.3 ± 12.4 cells/mm^2^ (IHC)	20.7 ± 8.1 cells/mm^2^	Cohen’s *d* = 3.02	<0.001
IL-6 level^§^	32.4 → 18.7 pg/mL (*Δ* = −42.3%)	30.1 → 27.5 pg/mL (*Δ* = −8.6%)	*β* = −0.58 (SE = 0.13)	0.002
ERα/PR crosstalk^‡^	ERα-PR co-expression ↓48% (IF)	No significant change	OR = 0.32 (95% CI 0.15–0.69)	0.006
Estrobolome function^†^	β-glucuronidase activity ↓37.9%	↓8.3%	*Δ* = −29.6%	0.008

*Values are presented as mean ± SD. ^†^Values are presented as median (IQR). ^‡^Categorical variables are presented as counts (percentages); ^§^values are assessed via methylation-specific PCR (MSP).

## 4 Discussion

In this study, we systematically evaluated the effect of metformin on the regulation of estrogen levels and prognostic nursing outcomes in LABC patients with T2DM. The results indicate that metformin effectively regulates hormone levels through multiple mechanisms and significantly improves survival outcomes. Specifically, metformin can lower serum estradiol levels by inhibiting aromatase activity and reducing the conversion of androgens to estrogen, and further reduce the bioavailability of estrogen by upregulating sex hormone binding globulin and improving the tumor microenvironment. Compared with conventional treatment, patients in the metformin group had significantly better blood glucose control, reduced glucose fluctuations, increased insulin sensitivity, and a lower incidence of hypoglycemic events. In terms of clinical nursing, the use of metformin promotes patient compliance with treatment, reduces the incidence of postoperative infections, and significantly shortens incision healing time. In addition, patients’ self-care ability, pain control effectiveness, and nursing time efficiency have all been improved, indicating that metformin has a synergistic effect on promoting nursing outcomes through metabolic stability. Regarding tumor prognosis, patients in the metformin group showed a significant improvement in 3-year OS rate, complete pathological remission rate, and CTC count. These results indicate that metformin significantly improves the tumor prognosis of LABC patients with T2DM not only by regulating hormone levels, but also by inhibiting tumor proliferation and metastasis.

Our findings are consistent with previous studies, indicating that metformin has a dual role in metabolic regulation and tumor suppression. The beneficial effects of metformin seem to go beyond blood glucose regulation. Mechanistically, it contributes to immune hormone metabolism crosstalk, promotes CD8^+^ T cell infiltration, inhibits inflammatory cytokines such as IL-6, and regulates estrogen metabolism through mechanisms such as ERα promoter methylation and p-Ser118 phosphorylation. In addition, the decrease in aromatase activity and improvement in estradiol group function indicate that metformin has systemic and tumor local endocrine regulatory effects. These findings emphasize the rationale of metformin as a drug that not only regulates systemic metabolism but also directly reshapes the tumor microenvironment to inhibit progression and metastasis.

Despite rigorous design, this study has several limitations. The retrospective nature of queues introduces inherent information bias, although PSM is used to alleviate confusion. And although a follow-up period of 36 months is sufficient for mid-term outcome evaluation, it may not capture long-term survival trends, adverse events, or drug tolerance. Third, the study did not stratify the efficacy according to cancer molecular subtypes. These factors deserve careful explanation and emphasize the need for expanded surveys targeting specific subtypes.

Prospective, multicenter randomized controlled trials with longer follow-up periods are crucial to effectively validate these findings. Future research should stratify patients by tumor subtype and include endpoints related to long-term efficacy, safety, and quality of life. Integrating precision medicine tools such as genomics, molecular imaging, and immune analysis can identify the subgroups most likely to benefit from metformin-based interventions. Mechanistically, multiple omics methods, including single-cell RNA sequencing, proteomics, and metabolomics, should be applied to elucidate the complex interactions between metformin, metabolic pathways, tumor immunity, and estrogen signaling. This study may help identify medication care strategies for patients and further advance the personalized treatment paradigm for tumor metabolic complications.
